# An animated depiction of major depression epidemiology

**DOI:** 10.1186/1471-244X-7-23

**Published:** 2007-06-08

**Authors:** Scott B Patten

**Affiliations:** 1 Department of Community Health Sciences, University of Calgary, 3330 Hospital Drive NW, Calgary, Alberta, T2N 4N1, Canada

## Abstract

**Background:**

Epidemiologic estimates are now available for a variety of parameters related to major depression epidemiology (incidence, prevalence, etc.). These estimates are potentially useful for policy and planning purposes, but it is first necessary that they be synthesized into a coherent picture of the epidemiology of the condition. Several attempts to do so have been made using mathematical modeling procedures. However, this information is not easy to communicate to users of epidemiological data (clinicians, administrators, policy makers).

**Methods:**

In this study, up-to-date data on major depression epidemiology were integrated using a discrete event simulation model. The mathematical model was animated in Virtual Reality Modeling Language (VRML) to create a visual, rather than mathematical, depiction of the epidemiology.

**Results:**

Consistent with existing literature, the model highlights potential advantages of population health strategies that emphasize access to effective long-term treatment. The paper contains a web-link to the animation.

**Conclusion:**

Visual animation of epidemiological results may be an effective knowledge translation tool. In clinical practice, such animations could potentially assist with patient education and enhanced long-term compliance.

## Background

A comprehensive picture of the epidemiology of an episodic condition cannot be embodied in a single parameter such as an estimate of incidence or prevalence. The incidence of major depressive disorder conveys information about the risk of onset of an initial episode, but this is of limited value for clinical practice (where presentations occur after onset) or for public policy (since the rate of onset of new episodes does not adequately reflect the burden of disease). Prevalence is in some respects an even more obscure parameter. Prevalence is the number or proportion ("prevalence proportion") of population members who have a disorder at a point ("point prevalence") in time or during a period ("period prevalence") in time. This parameter is salient to the problem of quantifying disease burden but ultimately reflects a balance between entry into the prevalence pool (incidence or recurrence) and a other factors such as episode duration and mortality.

Attempts to integrate epidemiological estimates into a comprehensive "picture" of the epidemiology of major depression have used Markov models [1,2] and micro-simulation approaches [3]. The complexity of major depression epidemiology has required the development of fairly complex models, the mathematics of which may not be of great interest to many users of such information. A solution may be found in the use of modeling techniques that can be linked to visual depictions of the population dynamics. In this way, complex information can be processed visually, rather than by using equations, transition matrices etc. In this project, a set of plausible estimates for key parameters were extracted from available sources, supplemented by information from the international literature. These were combined using a simulation model, and a visual depiction of the epidemiology was produced in the form of an animation.

In a previous study, data from a longitudinal study called the National Population Health Survey (NPHS) was used to develop a prognosis calculator for major depression [4]. This prior project was similar to the current one in its use of a discrete event simulation modeling approach, but the previous analysis only examined the issue of episode duration. The current project uses the best available data from the literature rather than using data from a single survey source. It also attempts to integrate the various estimates into a more comprehensive description of the epidemiology.

## Methods

### Selection of epidemiological calibration targets for the simulation model

A set of values for key epidemiological parameters were selected in the development of the simulation model, based largely on existing literature. The objective was not to calibrate the model to a particular data set, but rather to use a set of reasonable values for the key parameters. Appendix I, see Additional file 1, presents more detailed information on the selection of specific parameter values. A summary is presented in Table 1. In brief, lifetime prevalence was assumed to be approximately 20% for men and 30% for women, estimates put forward by Kruijshaar et al., taking into account the recall bias that almost certainly effects such estimates [3]. In some respects, these values may seem controversial, because they are higher than usually cited values, e.g. see a systematic review by Waraich et al. [5]. Andrews provides a review of evidence that the lifetime prevalence is probably much higher than has been traditionally believed [6]. The duration of episodes was assumed to follow a pattern whereby the longer an episode lasts, the lower the probability of recovery. A similar pattern was assumed, based on the literature, for episode-free intervals. The longer an episode of remission lasts, the lower the recurrence frequency. Again, this is consistent with Kruijshaar et al.'s work [3], which used lognormal and Weibull distributions to model these effects. More information about the basis for these assumptions is contained in Appendix I, see Additional file 1. The model also included entry into the population at risk and age-specific mortality risks reflecting those of the general population. Mortality was assumed to be increased in people who have had a major depressive episode, but only slightly so, with the relative risk for mortality being 1.1, a 10% increase relative to the general population. Again, additional detail about the selection of these parameters is in Appendix I, see Additional file 1.

**Table 1:** Targets for model calibration

	Literature synthesis	Modeling strategy
Incidence (annual)	High in teenagers – approximately 7.1% in female adolescents, 4.4% in male adolescents. Declines with age.	Use of a Weibull distribution, to reflect declining incidence with age. Separate scale and shape parameters for men and women. Calibrate first incidence to result in lifetime prevalence of approximately 20% for men and 30% for women.
Point prevalence	Overall, approximately 2%. Higher in women than in men, declines with age.	Relative risk of recurrence selected (see below) to predict the point prevalence.
Recurrence	Approximately 10% in first year after recovery from an initial episode. Approximately 85% will have at least one recurrence during their lifetime. Recurrence rate higher after multiple episodes than single episodes.	First recurrence simulated with a Weibull distribution, men and women combined together with a single scale and shape parameter. Relative risk for multiple recurrence was estimated representing a higher risk among those with prior episodes. The relative risk for recurrence was calibrated to achieve a point prevalence of 1.8% holding the Weibull parameters for first incidence and episode duration constant.
Episode duration	Approximately 15% report a 2 week duration, the time at which the diagnosis is technically possible. However, a similar proportion is not recovered after 2 years.	Weibull distribution, a single scale and shape parameter for men and women. Model calibrated by matching the simulated proportion with a two week and 2 year duration at 15% and 85%, respectively.

### Simulation strategy

The simulation model was programmed in software called Arena [7]. This software provides a graphical interface for development of SIMAN models using discrete event simulation. The model was set up so that simulated subjects or "entities" enter at age 15. Following Kruijshaar [3], the simulation began by assignment of a death date to each simulated entity (depicting a person), based on sex and age-specific mortality data for Canada, which was downloaded from the Statistics Canada website using an on-line data access utility called E-STAT [8]. Next, an incidence date was simulated using a cumulative Weibull distribution. This distribution was selected to reflect the expected decline in incidence with advancing age (see Additional file 1). When the incidence date occurred before the death date, an episode would occur during that subject's lifetime, and in this case the death date was recalculated using a relative risk for mortality of 1.1. A meta-analysis by Wulsin et al. [9] estimated the relative risk at 1.8, but this is almost certainly an overestimate as most of the studies included in the meta-analysis were clinical studies. A program called OptQuest, which is a part of the Arena package, was used to identify Weibull parameters (shape and scale) for incidence that were consistent both with estimates for the adolescent age group (which have been found to be very high in some studies, see Additional file 1), but diminishing at a sufficient rate to be consistent with the selected values for lifetime prevalence. OptQuest conducts a series of Monte Carlo simulations in order to identify parameters that closely fit modeling objectives.

A similar strategy was employed to simulate other elements in the model. Weibull distributions for episode duration, and the duration of remitted periods between episodes were identified. After resolution of an initial episode, the model entities moved into a first remission state. This approach resembles that of Kruijshaar et al. [3], except that Kruijshaar et al. [3] used lognormal distributions to describe episode duration. Previous work has determined that the Weibull distribution can also provide a good description [4]. OptQuest was used to find Weibull parameters for episode duration consistent with the selected calibration targets: approximately 15% recovered before three weeks, but approximately 15% are still not recovered after 2 years, see Appendix I for a more detailed discussion. Once appropriate values for these Weibull parameters were found, the relative risk of recurrence was sought, again using OptQuest, and calibrated to a targeted point prevalence of 1.8% [10].

In the model, when the simulated death date was reached, the entity left the simulation. Otherwise, subjects with depressive episodes continue to cycle between depressed and remitted states until their death date is reached. Eventually, a balance between entry and exit (death) is reached, and the various calibration parameters assume steady state values. In order to reach this state, the model was run for 250,000 days. This steady state allowed the model to be calibrated, using OptQuest as described above.

### Making a visual animation

Arena can save the results of a simulation model into a file type (which Arena calls a "playback" file) that can be imported into a Virtual Reality Modeling Language (VRML) program and animated in three dimensions using a program called the Arena 3-D player. Video can be captured from the animation. The animations presented in this paper were captured as "avi" files, edited with addition of captions in a program called "Camtasia" and then saved as MacroMedia Flash files. These files were uploaded to the University of Calgary's Dspace under a Creative Commons license for open access. The Flash movie contains three parts. In the first part, the structure of the visual animation is presented. In the second part, the fully calibrated model is depicted. In the third section the model is animated with the recurrence pattern in subjects with multiple episodes having been changed to that of subjects with single episodes. The third part is a way of depicting the population health impact of highly effective tertiary prevention, ie. long-term treatment substantially reducing the relapse rate. The simulations were run at 500,000 times "real time." This means that each second in the simulation represents about six days of time. The rate of entry into the population was set so that approximately 1000 simulated people (entities) would be present in the simulation at steady state. In order that subjects could be seen moving from state to state, transitions were set to last for two weeks in simulation time. The two week transition time seems clinically reasonable, and avoids a problem that would arise in the animations if the transitions occurred too quickly to be seen easily.

## Results

Table 2 shows optimized parameters for the simulation model. The time units for the distributions are days. The Weibull parameters themselves are not readily interpretable. The cumulative Weibull distribution of time to a first episode, which was used, for example, to depict first incidence in these simulations is 1 – exp [-(time/a)^b^], where 'a' is the scale parameter and 'b' is the shape parameter. From these Weibull parameters, 'a' and 'b' it is possible to calculate age specific incidence rates as: b/a * (t/a)^b-1^. Here, time is in days with time = 0 being the time of entry into the model at age 15, so that time specific incidence can be rescaled to represent age specific incidence. Similarly, scale and shape parameters for episode duration, duration of first remission and duration of remission after multiple recurrences can be expressed more understandably as time specific recovery and recurrence rates.

**Table 2:** Optimized estimates: Weibull distributions*

Model parameter:	Scale	Shape
Incidence (Men)	400000	0.45
Incidence (Women)	130000	0.425
First recurrence	8225.5	0.782
Episode duration	205	0.521

* Relative risk for recurrence: 2.75

**Figure 1:**
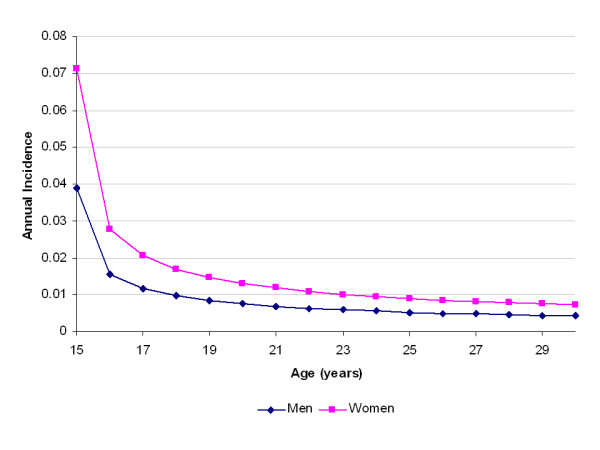
Age-specific incidence rates from the calibrated simulation model.

**Figure 2:**
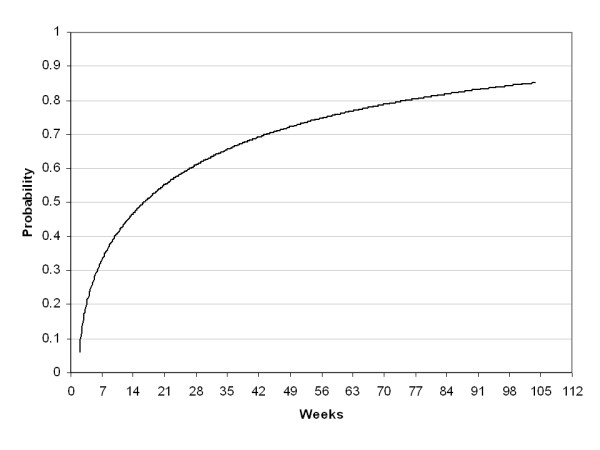
Cumulative probability of recovery.

**Figure 3:**
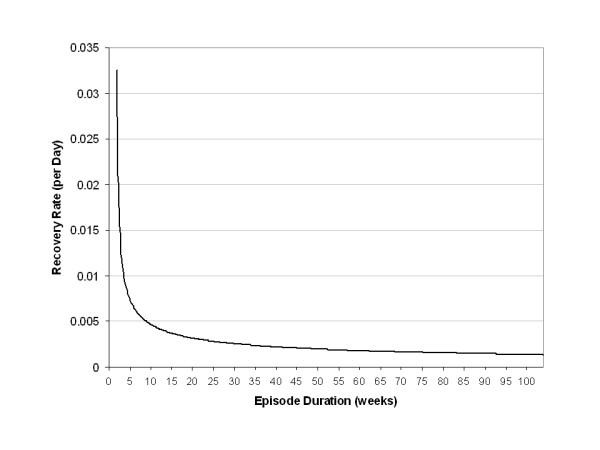
Recovery rate, by episode duration.

**Figure 4:**
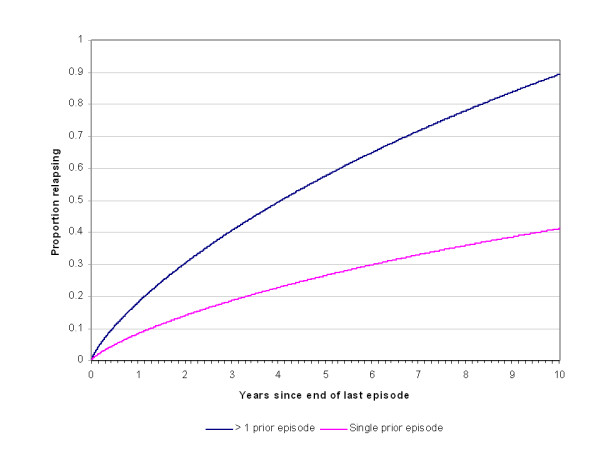
Cumulative frequency of relapse, by time since last episode.

The parameters for incidence translate into an average annualized incidence at age 16 (t = 365 days) of 1.7% for men and 4.0% for women. By age 25 (t = 3650) these rates decline to less than 1%. The incidence rates continue to decline with age, as depicted in Figure 1. The recovery pattern is depicted in Figure 2, as the cumulative proportion of subjects recovered from an episode over time. Figure 3 shows the corresponding recovery rates, rather than the cumulative proportion recovered. Figure 4 presents the cumulative probability of relapse after single and recurrent episodes.

The Dspace link that connects to the Flash video file may be found here [11]. A dramatic reduction in point prevalence is seen in association with effective tertiary prevention, because of a lower proportion of subjects in the recurrent episode states in a recurrence at any point in time. Two longer simulation intervals (each about 2.5 minutes) can be found here [12]. These again present the basic calibrated model (first) followed by the depiction of effective tertiary prevention (second), except that an Arena "dashboard" has been added, so that key model parameters are monitored on the bottom of the screen. The simulations need to be fairly long, because the animations reflect stochastic fluctuations in the model outputs. Mean values for n = 100 simulations are presented in Table 3. Table 3 also presents the calibrated model with a relative risk for mortality of 1.8 (centre column). As expected, the higher mortality in depression leads to slightly lower point prevalence, but most of the parameters are very similar. A set of n = 100 replications of the model with effective tertiary prevention is also presented in Table 3 (right column). Here, effective tertiary prevention is defined as a reduction in the recurrence rate for people with highly recurrent (≥ 3 episodes) disorders to the same recurrence rate as those who have had only one past episode.

**Table 3:** Mean values for model parameters (%), after N = 100 replications

	Main calibration	Mortality sensitivity analysis*	Strong tertiary prevention^†^
Incidence at age 15 (♂)	4.2%	4.1%	4.2%
Incidence at age 15 (♀)	8.2%	8.1%	8.2%
Total lifetime prevalence	25.1%	23.3%	25.1%
Lifetime prevalence (♂)	18.5%	16.9%	18.6%
Lifetime prevalence (♀)	31.5%	29.7%	31.6%
Point prevalence	2.4%	2.1%	1.5%
Episodes lasting < 3 weeks	15.8%	15.8%	15.7%
Episodes lasting ≥ 2 years (%)	14.8%	14.8%	14.7%
Proportion MDD with single episode	16.9%	19.5%	16.9%
Lifetime morbidity proportion**	34.0%	34.2%	34.0%
Proportion relapse within 1 year	10.0%	10.5%	10.0%

* Relative risk for mortality assumed to be 1.8 rather than 1.1 in main calibration. See Appendix II for a more comprehensive set of simulations.

** the model records the number of dying entities who have had an episode during their time in the population at risk.

^† ^Relative risk for recurrence = 1. Risk of recurrence after multiple episodes equal to that after single episodes. N = 100 model replications. See Appendix II for a more comprehensive set of simulations.

As expected, mortality has an effect on point prevalence, but the large change in associated mortality has only a small effect. On the other hand, changing the recurrence rate has a large effect on point prevalence.

### Sensitivity analyses

A variety of analyses linking model variables (e.g. scale and shape parameters from the Weibull distributions) to incidence, recurrence and prevalence are presented in Appendix II, see Additional file 2. This Appendix also contains a series of simulations, each with n = 25 replications showing the effect on point prevalence of alteration to various model parameters.

## Discussion

The simulation model presented represents a crude synthesis of available data about the epidemiology of major depression. Rather than accept lifetime prevalence estimates made directly from epidemiological studies, thereby ignoring evidence of recall bias that is likely to affect such estimates [13], an estimate of the probable lifetime prevalence based on a combination of incidence and prevalence data from Kruijshaaar [3] was used. The mortality estimate is also somewhat arbitrary. Prior studies pointed towards a higher relative risk for mortality in subjects with major depression, but these are almost certainly over-estimates, as their samples consisted of hospitalized clinical subjects, see Additional file 1 (Appendix I). However, the estimate adopted here is somewhat arbitrary. A sensitivity analysis does not indicate that this makes a large difference to the simulation model, see Additional file 2 (Appendix II). Similarly, values for episode duration and recurrence rates that were used to calibrate the module represent reasonable values given published estimates. The rates of recovery from episodes as depicted here are lower than those reported from a Canadian data source, the National Population Health Survey [4]. However, this study used a brief diagnostic instrument which may have picked up some sub-clinical cases [14].

Despite these limitations, an understanding of certain key elements of major depression epidemiology has solidified during the past decade: recurrence is greater than first incidence, incidence is high in young people, and declines with age, the recovery and remission rates are time dependent. The model reflects all of these elements of the condition. The model presents a visual impression of the interaction between a reasonable set of assumptions about the epidemiology of the condition.

## Conclusion

The epidemiology of major depression is complex, and models that attempt to reflect this complexity may have a diminished impact because of their own inherent complexity. Depiction of a simulation model as a visual animation may assist with communicating its implications to a broad audience. Animations of this sort may also be helpful for patient education, and enhanced compliance as they illustrate some of the evidence for therapeutic recommendations. The results presented here are consistent with a conception of major depression as a chronic, recurrent condition [15] and suggest that relevant long-term strategies can have a large impact on population health status.

## Competing interests

The author(s) declare that they have no competing interests.

## Pre-publication history

The pre-publication history for this paper can be accessed here:



## Supplementary Material

Additional file 1 Click here for file

Additional file 2 Click here for file
