# Supplementary material for: An animated depiction of major depression epidemiology
Source: BMC Psychiatry. 2007 Jun 8;7:23. doi: 10.1186/1471-244X-7-23 (PMC1906857; doi:10.1186/1471-244X-7-23)
Supplement: Additional file 1 [file 1471-244X-7-23-S1.pdf]

## Appendix I: Selection of Calibration Targets for the Model

Note: a tabular summary is presented in Table 2 in the main manuscript.

*Incidence*

Historically, estimates of major depression incidence derived from large prospective studies, such as the Lundby Study [1], the Stirling County Study [2] and the Baltimore Epidemiologic Catchment Area (ECA) follow-up [3]. These studies have reported incidence rates between 2.0 per 1000/year and 4.5 per 1000/year, and have generally observed higher rates in women than in men. More recently, several studies have reported much higher incidence rates. The Finnish ODIN study [4] produced an estimate of 28.5 per 1000/year (approximately 3%), and the Netherlands NEMESIS study [5] reported 2.72 per 100 person-years [6]. These studies used lifetime assessment interviews at baseline to determine who was at risk for a first episode, an approach that has been shown not to be accurate [7,8], so the higher estimates probably mean that these were not truly first incidence studies, but rather included some recurrent cases. Another complicating factor is that several studies have reported very high incidence rates in adolescent samples [9,10]. In these studies, the cumulative incidence over a few years was of similar magnitude to lifetime prevalence estimates from adult community samples. In high school aged adolescents, Lewinsohn et al. reported a one year first incidence of major depression of 7.1% in females and 4.5% in males [10]. Very high incidence in the 12-24 year old age group has also been observed in Canada [11].The high incidence in adolescents must decline with advancing age so that the lifetime

prevalence produced by the model would not be excessively high. The targets for model calibration in the initial year were those from Lewinsohn: 7.1% annually in women and 2.9% in men [10] at the time of entry to the model along with Kruijshaar et al.'s estimate of lifetime prevalence at 30% in women and 20% in men [12].

*Point Prevalence*

Point prevalence is formally defined as the frequency of disease in a population at a point in time. In psychiatric epidemiology, point or current prevalence usually means a brief period prevalence, typically 30 days. A 1994 review of the historical literature by Wittchen et al. placed the point prevalence of major depression at about 3% [13]. In the NEMESIS study, the one month prevalence was 3.4% for women and 1.9% for men [14]. Murphy et al, placed this parameter within the range of 2.2% to 2.6% [15], citing estimates from the Stirling County Study, ECA Study [16] and an Edmonton study [17]. As such, there is fairly consistent evidence that approximately 2-3% of the population have major depression at any one point in time. In the Canadian Community Health Survey: Mental Health and Wellbeing (CCHS 1.2), with exclusion of apparent bipolar cases, 1.8% of respondents had a major depressive episode in the past 30 days [18].

*Lifetime Prevalence*

The two most widely cited estimates are those from the National Comorbidity Survey (17.1%) [19] and its replication (16.2%) [20]. The CCHS 1.2 in Canada reported

a 12.2% lifetime prevalence [18], similar to the 12.8% figure reported by the European ESEMeD studies [21]. Given evidence of recall failure with historical assessment [7,12], these are probably all underestimates, an hypothesis that has received support from studies using modeling approaches [12]. A lifetime prevalence of 32% in high school aged women and 16% in high school aged men was reported by Lewinsohn et al. [10]. An estimate of this type may have reduced susceptibility to recall bias since the episodes evaluated occurred in greater temporal proximity to the interview. This idea is consistent with Kruijshaar's assertion that lifetime prevalence is probably higher than retrospective estimates suggest: she estimated that it probably falls in the range of 30% to 20% respectively in women and men [12].

*Mortality*

The impact of major depression on mortality has mostly been investigated by studies that followed clinical subjects over time. Such estimates may not be generalizable to community populations. As such, the relative risk for mortality of 1.8 arising from a systematic review conducted by Wulsin et al. [22] is probably an over estimate. For this reason, a somewhat arbitrary value of 1.1 was selected as a calibration target for the visual simulation. However, a sensitivity analysis was carried out, see Appendix II and Table 3 in the paper.

*Recurrence*

In the 12-14 year follow-up of the Baltimore ECA studies, approximately 50% of subjects who had lifetime major depression had a recurrence during the follow-up interval [3]. There was no sex difference in the recurrence rate, but recurrent episodes, on average, were found to be briefer than initial episodes. The National Collaborative Depression Study [23] found a higher rate of recurrence in women. A proviso is that the National Collaborative Study did not use a general population sample. In this study, recovered subjects had an 85% chance of having a recurrence during the following 15 years. Furthermore approximately half of subjects who remained well for five years after recovery had a relapse within the next 10 years. In a more highly recurrent group, the same study found that 41% relapsed within a year, and 74% within 5 years [24]. These figures provide an indication that the rate of recurrence changes over time, with increasing time since recovery being associated with a diminished probability of recurrence.

The Canadian National Population Health Survey prospectively follows a cohort of Canadians who were randomly selected from the population in 1994 and 1995. They are interviewed every two years with an interview that includes the CIDI Short Form for Major Depression [25]. Of 8368 subjects with no episodes of major depression in the first four interview cycles, 175 had an episode in the final cycle – a frequency of approximately 2%. Subjects with one episode in the previous four cycles (n=935), 108 had an episode in the final cycle, an incidence of approximately 12%. Of 303 subjects with more than one episode in the first four cycles, 105 had one in the final cycle,

approximately one third. These results are consistent with the idea that the probability of recurrence increases with successive episodes.

*Episode Duration*

Data from the NEMESIS study provide the best available data on episode duration, and detailed descriptive analyses have been presented by Spijker [26,27]. This prospective study included interviews at three time points. NEMESIS included a Life Chart Interview to determine episode duration. Analyses of the NEMESIS data indicated that many episodes are brief, the median duration being reported as 3 months, whereas a smaller number of episodes were much longer, so that the mean duration was 8.4 months (with an upper limit of 24 months imposed due to the study design). The proportion of episodes lasting two years or longer was estimated as being approximately 20% [26]. This pattern was described using a lognormal distribution in modeling studies by Vos et al. [28], who included data from several international studies including that of Lewinsohn et al. [29] and Eaton et al. [3]. The probability of remission in the studies reviewed was higher than that of NEMESIS in these studies, and was approximately 90%. The Canadian National Population Health Survey (NPHS) included questions on episode duration, and Markov models based on the NPHS data predicted a one-year recovery rate in the range of 95% [30,31]. Consistent with the pattern seen in the Vos et al. review, the proportion reporting symptom durations of 2-3 weeks (with 2 weeks being the minimum duration in DSM-IV) was high, in the range of 15-20% of episodes in the NPHS [32]. However, the NPHS used a brief version of the CIDI [25], which may have detected

some sub-clinical episodes. The NEMESIS project may be the most credible source of episode duration data, but 80% recovery after two years is more pessimistic than all other studies. Based in the literature, a pattern characterized by approximately 15% recovery after 3 weeks of symptoms, but where 15% remained depressed after two years seemed to be a plausible set of values for calibration targets for the model.

Reference List


(1) Hagnell O, Lanke J, Rorsman B, Ojesjo L. Are we entering an age of melancholy? Depressive illnesses in a prospective epidemiological study over 25 years: the Lundby Study, Sweden. Psychol Med 1982;12:279-89.

(2) Lehtinen V, Veijola J, Lindholm T, Moring J, Puukka P, Väisänen E. Incidence of mental disorders in the Finnish UKKI Study. Br J Psychiatry 1996;168:672-8.

(3) Eaton WW, Anthony JC, Gallo J, Cai G, Tien A, Romanoski A, et al. Natural history of diagnostic interview schedule/DSM-IV major depression. The Baltimore epidemiological catchment area follow-up. Arch Gen Psychiatry 1997;54:993-9.

(4) Lehtinen V, Sohlman B, Nummelin T, Salomaa M, Ayuso-Mateos J-L, Dowrick C. The estimated incidence of depressive disorder and its determinants in the Finnish ODIN sample. Soc Psychiatry Psychiatr Epidemiol 2005;40:778-84.

(5) Bijl RV, van Zessen G, Ravelli A, de Rijk C, Langendoen Y. The Netherlands Mental Health Survey and Incidence Study (NEMESIS): objectives and design. Soc Psychiatry Psychiatr Epidemiol 1998;33:581-6.

(6) Bijl RV, de Graaf R, Ravelli A, Smit F, Vollebergh WA. Gender and age-specific first incidence of DSM-III-R psychiatric disorders in the general population. Results from the Netherlands Mental Health Survey and Incidence Study (NEMESIS). Soc Psychiatry Psychiatr Epidemiol 2002;(37):372-9.

(7) Andrews G, Anstey K, Brodaty H, Issakidis C, Luscombe G. Recall of depressive episode 25 years previously. Psychol Med 1999;29:787-91.

(8) Newman SC, Bland RC. Incidence of mental disorders in Edmonton: estimates of rates and methodological issues. J Psychiatr Res 1998;32:273-82.

(9) Oldehinkel AJ, Wittchen H-U, Schuster P. Prevalence, 20-month incidence and outcome of unipolar depressive disorders in a community sample of adolescents. Psychol Med 1999;29:655-68.

(10) Lewinsohn PM, Hops h, Roberts RE, Seeley JR, Andrews JA. Adolescent psychopathology: I. Prevalence and incidence of depression and other DSM-III-R disorders in high school students. J Abn Psychol 1993;102:133-44.

(11) Patten SB. Major depression incidence in Canada. Can Med Assoc J 2000;163:714-5.

(12) Kruijshaar ME, Barendregt J, Vos T, de Graaf R, Spijker J, Andrews G. Lifetime prevalence estimates of major depression: An indirect estimation method and a quantification of recall bias. Eur J Epidemiol 2005;20:103-11.

(13) Wittchen H-U, Knäuper B, Kessler RC. Lifetime risk of depression. Br J Psychiatry 1994;165(suppl. 26):16-22.

(14) Ormel J, Oldehinkel AJ, Nolen WA, Vollebergh W. Psychosocial disability before, during and after a major depressive episode. A 3-wave population-based study of state, scar and trait effects. Arch Gen Psychiatry 2004;61:387-92.

(15) Murphy JM, Laird NM, Monson RR, Sobol AM, Leighton AH. A 40-year perspective on the prevalence of depression. Arch Gen Psychiatry 2000;57:209-15.

(16) Regier DA, Boyd JH, Burke JD, Myers JK, Kramer M, Robins LN, et al. One-month prevalence of mental disorders in the United States. Based on five epidemiological catchment area sites. Arch Gen Psychiatry 1988;45:977-86.

(17) Spaner D, Bland RC, Newman SC. Major Depressive Disorder. Acta Psychiatr Scand 1994;Suppl 376:7-15.

(18) Patten SB, Wang JL, Williams JVA, Currie SR, Beck CA, Maxwell CJ, et al. Descriptive epidemiology of major depression in Canada. Can J Psychiatry 2006;51:84-90.

(19) Kessler RC, McGonagle KA, Zhao S, Nelson CB, Hughes M, Eshleman S, et al. Lifetime and 12-month prevalence of DSM-III-R psychiatric disorders in the United States. Results from the National Comorbidity Survey. Arch Gen Psychiatry 1994;51:8-19.

(20) Kessler RC, Berglund P, Demler O, Jin R, Koretz D, Merikangas KR, et al. The epidemiology of major depressive disorder: results from the National Comorbidity Survey Replication (NCS-R). JAMA 2003;289:3095-105.

(21) ESEMeD/MHEDEA 2000 Investigators. Prevalence of mental disorders in Europe: results from the European Study of the Epidemiology of Mental Disorders (ESEMeD) project. Acta Psychiatr Scand 2004;109(Suppl. 420):21-7.

(22) Wulsin LR, Vaillant GE, Wells VE. A systematic review of the mortality of depression. Psychosom Med 1999;61:6-17.

(23) Mueller TI, Leon AC, Keller MB, Solomon DA, Endicott J, Coryell W, et al. Recurrence after recovery from major depressive disorder during 15 years of observational follow-up. Am J Psychiatry 1999;156:1000-6.

(24) Solomon DA, Keller MB, Leon AC, Mueller TI, Lavori PW, Shea MT, et al. Multiple recurrences of major depressive disorder. Am J Psychiatry 2000;157:229-33.

(25) Kessler RC, Andrews G, Mroczek D, Ustun B, Wittchen HU. The World Health Organization Composite International Diagnostic Interview Short-Form (CIDI-SF). Int J Methods Psychiatr Res 1998;7:171-85.

(26) Spijker J, de Graaf R, Bijl RV, Beekman ATF, Ormel J, Nolen WA. Duration of major depressive episodes in the general population: results from the Netherlands Mental Health Survey and Incidence Study (NEMESIS). Br J Psychiatry 2002;181:208-13.

(27) Spijker J, de Graaf R, Bijl RV, Beekman ATF, Ormel J, Nolen WA. Determinants of persistence of major depressive episodes in the general population. Results from the Netherlands Mental Health Survey and Incidence Study (NEMESIS). J Affect Disord 2004;81:231-40.

(28) Vos T, Haby MM, Berendregt JJ, Kruijshaar M, Corry J, Andrews G. The burden of major depression avoidable by longer-term treatment strategies. Arch Gen Psychiatry 2004;61:1097-103.

(29) Lewinsohn PM, Clarke GN, Seeley JR, Rohde P. Major depression in community adolescents: age at onset, episode duration, and time to recurrence. J Am Acad Child Adolesc Psychiatry 1994;33:809-18.

(30) Patten SB, Lee RC. Describing the longitudinal course of major depression using Markov models: Data integration across three national surveys. Popul Hlth Metr 2005;3:11.

(31) Patten SB, Lee RC. Refining Estimates of Major Depression Incidence and Episode Duration in Canada using a Monte Carlo Markov Model. Med Decis Making 2004;24:351-8.

(32) Patten SB. A major depression prognosis calculator based on episode duration. Clin Pract Epidemiol Mental Hlth 2006;2:13.
