# Supplementary material for: An animated depiction of major depression epidemiology
Source: BMC Psychiatry. 2007 Jun 8;7:23. doi: 10.1186/1471-244X-7-23 (PMC1906857; doi:10.1186/1471-244X-7-23)

Appendix II: Effect of Model Parameters on Simulated Prevalence

Incidence

In the model, incidence is not depicted as a single rate, but as a rate that changes with age. This is reflected by two Weibull parameters, a scale and shape parameter. A larger scale parameter can be interpreted as a longer time to event at the baseline time (upon entry into the population at risk), so that smaller scale parameters translate into higher age-specific incidence rates. This is depicted in Figures 1 and 2. Age-specific incidence curves for a variety of scale parameters are presented, all at the best-fitting values for the best fitting shape parameters (0.45 for men and 0.425 for women).

Figure 1. Annual Incidence for Men from Weibull Distributions

(Shape Parameter = 0.45), by Scale Parameter Values.

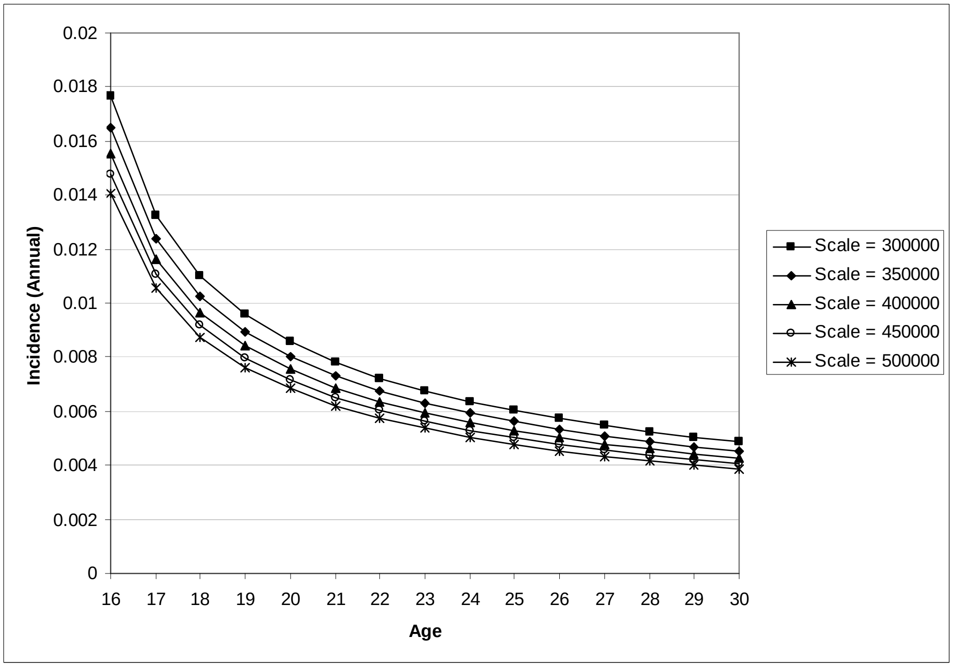

Figure 2. Annual Incidence for Women from Weibull Distributions

(Shape Parameter = 0.425), by Scale Parameter Values.

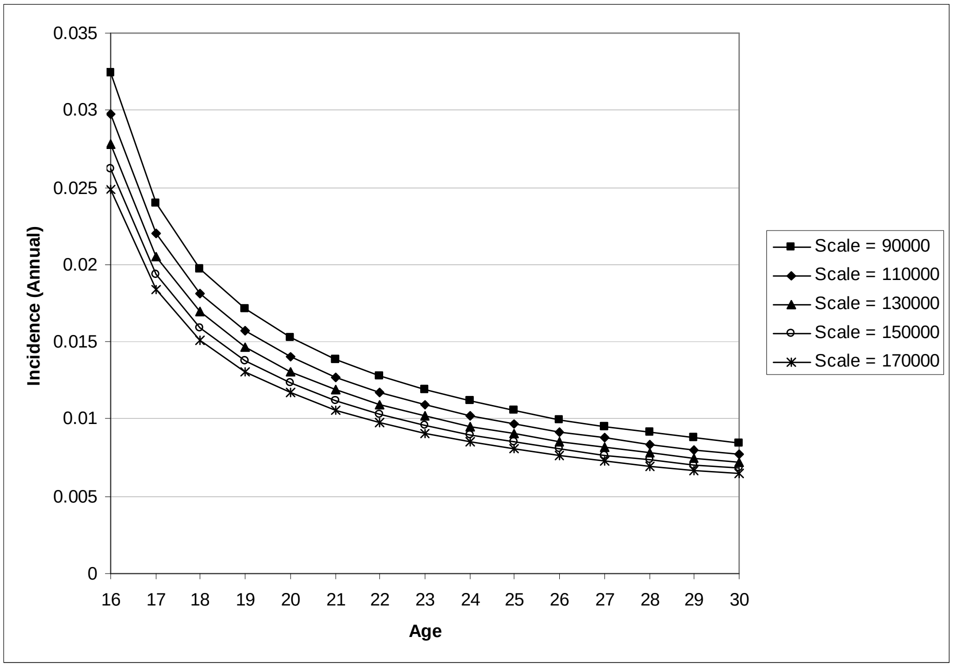

In the model, incidence is represented by a continuous distribution. The age specific incidence estimates in these figures represent the average daily incidence during the year in question, converted to an annual rate[1] for ease of interpretation.

Figure 3 presents a series of simulations of point prevalence at various scale parameter values in men, keeping all other parameters in the model stable. The values depicted are mean values for point prevalence over 25 replications with the model. In keeping with expectation, since smaller scale values are associated with higher age-specific incidence rates, larger values for the scale parameters are associated with a decline in point prevalence. The same pattern is seen in women, see Figure 4.

[1] Annual Incidence = 1 – exp(-daily rate*365)

Figure 3. Simulated Point Prevalence in Men, by Scale Parameter for the Weibull Parameter Depicting Incidence (Shape Parameter = 0.45).

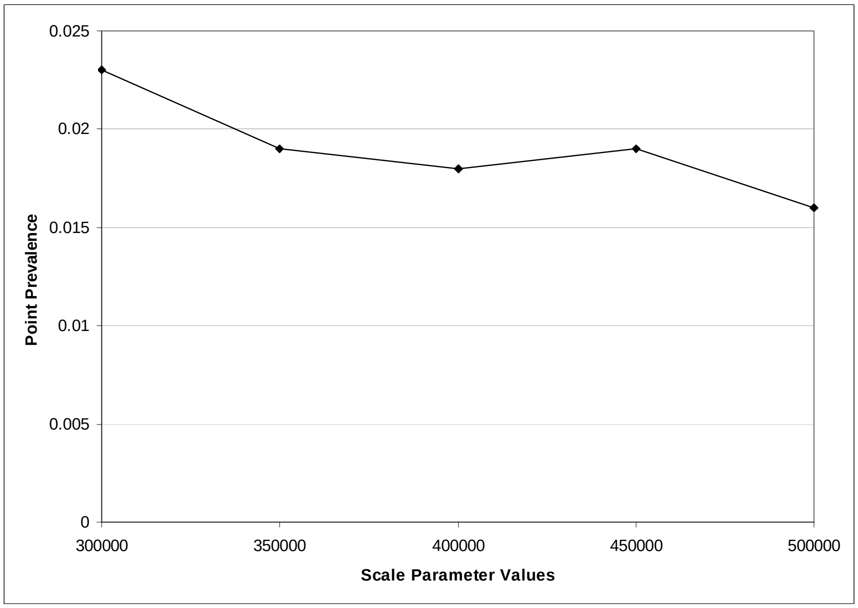

Figure 4. Simulated Point Prevalence in Women, by Scale Parameter for the Weibull Parameter Depicting Incidence (Shape Parameter = 0.425).

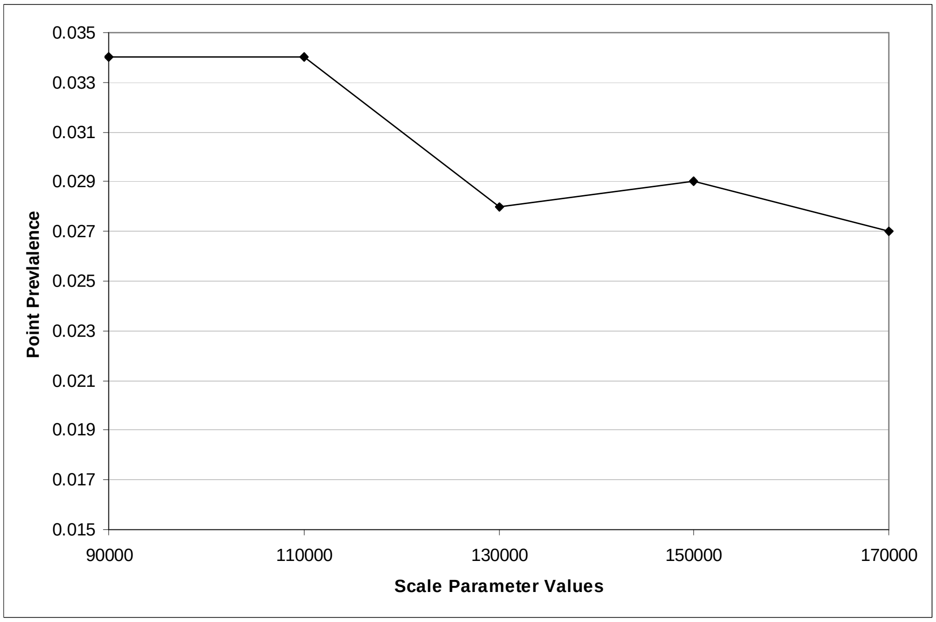

The shape parameter reflects the rate of decline in the incidence rate over time. Figures 5 and 6 present age-specific incidence at the best fitting scale parameters for men and women, respectively, using a variety of shape parameters.

Figure 5. Annual Incidence for Men from Weibull Distributions (Scale Parameter = 400000), by Shape Parameter.

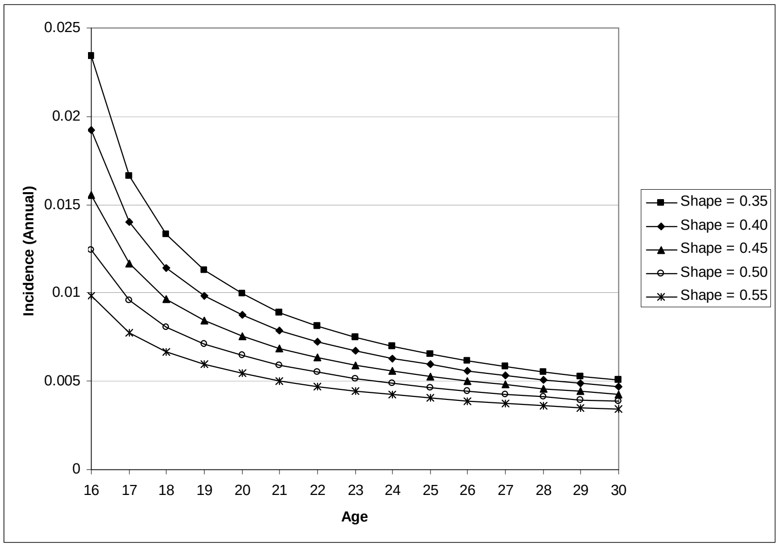

Figure 6. Annual Incidence for Women from Weibull Distributions (Scale Parameter = 130000), by Shape Parameter.

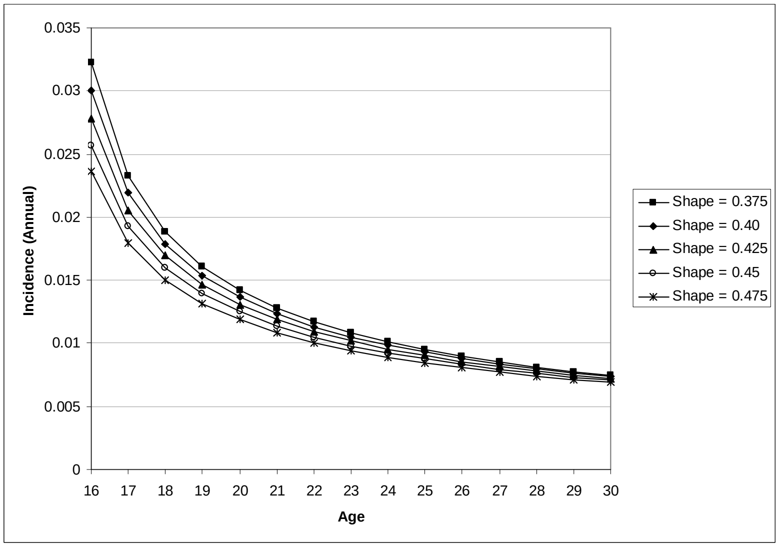

Point prevalence simulations for a variety of shape parameter values based on n=25 replications are depicted for men and women in Figures 7 and 8. The plots suggest a slight decline in simulated prevalence with increasing values for the shape parameter.

Figure 7. Simulated Point Prevalence for Men, by Shape Parameter (Scale Parameter = 400000).

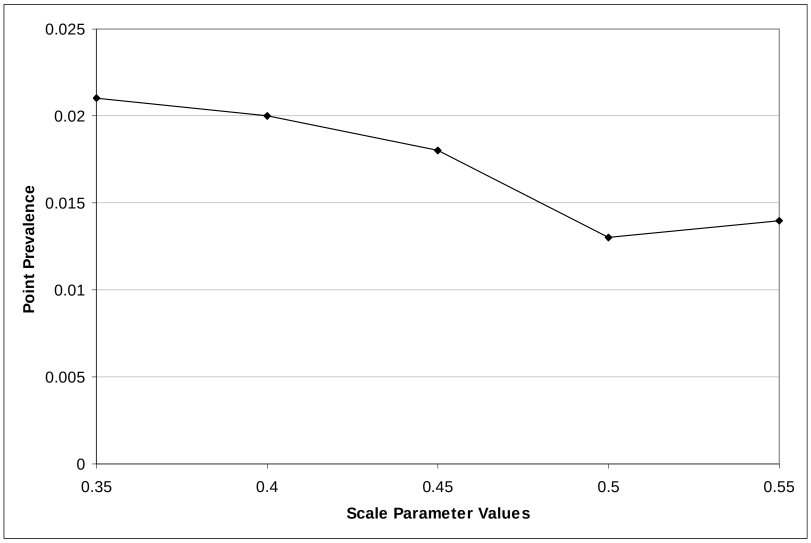

Figure 8. Simulated Point Prevalence for Women, by Shape Parameter (Scale Parameter = 130000).

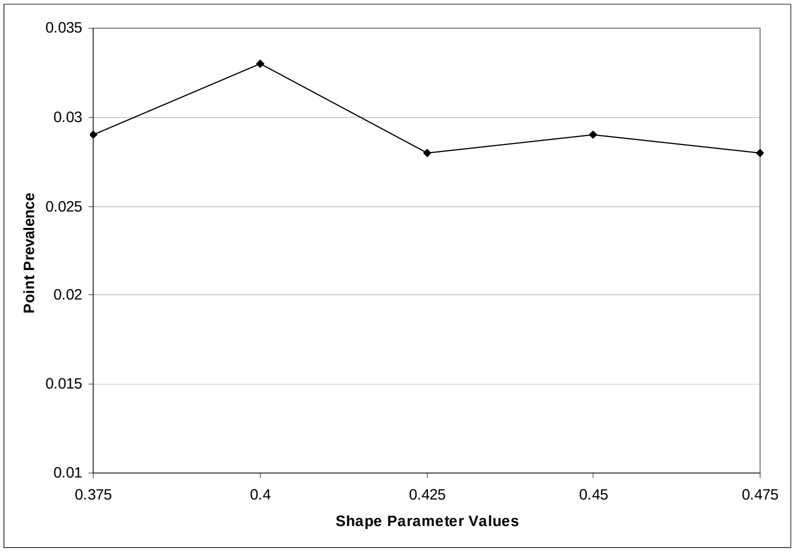

First Recurrence

Recurrence is expected to occur at higher rates than first incidence. Also, the distribution of time to recurrence is referenced to the time of recovery from the first episode. Figure 9 shows a series of simulations of point prevalence using increasing scale parameter values for the Weibull distribution depicting first recurrence, keeping the shape parameter constant for each simulation. Each point on the Figure represents the mean value of 25 replications.

Figure 9. Simulated Point Prevalence, by Scale Parameter (Shape Parameter = 0.782).

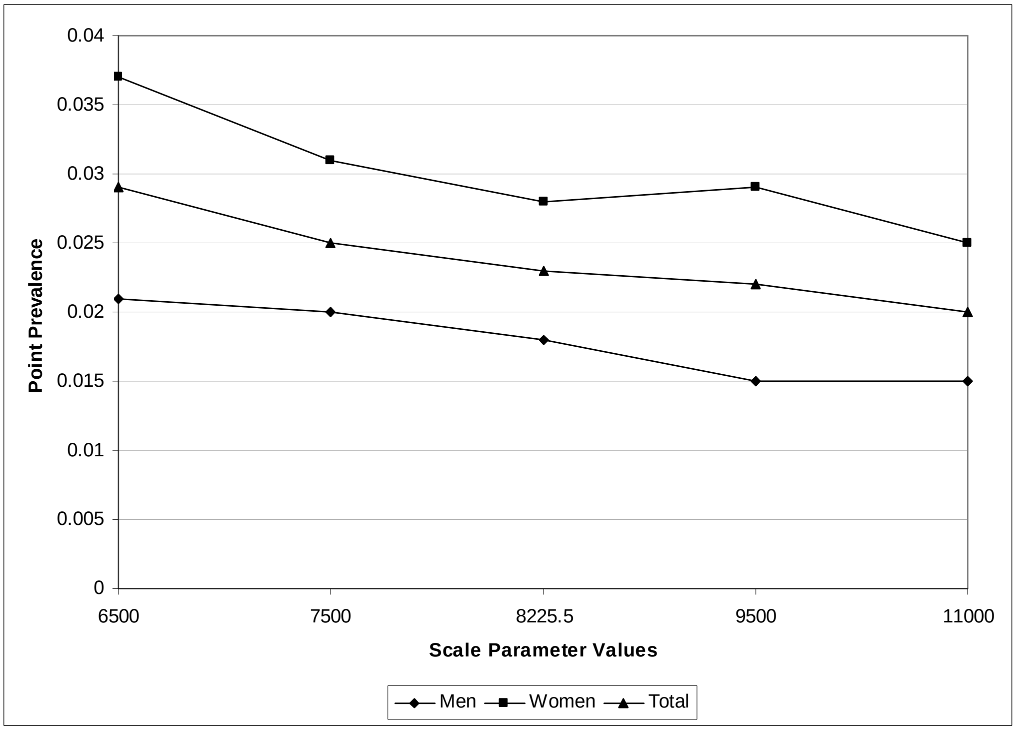

Increasing values of the shape parameter describe a less rapid decline in the first recurrence rate with time since recovery from an initial episode. For this reason, increasing values for this shape parameter may be expected to lead to increased point

prevalence, as is confirmed by a series of simulations (25 replications for each data point) presented in Figure 10.

Figure 10. Simulated Point Prevalence, by Shape Parameter for First Recurrence Rates (Scale = 8225.5).

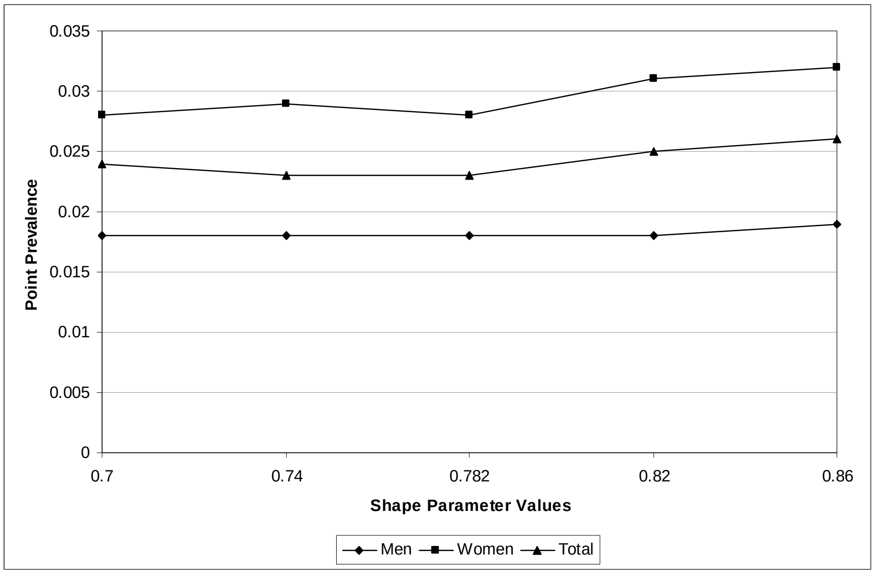

Multiple Recurrences

In the simulation model, the multiple recurrence state is defined as ≥ 3 episodes. The relative risk for recurrence in this state is expected to be strongly related to point prevalence in the population. Figure 11 presents a series of simulations (25 replications at each set of values) for point prevalence at four different values for the relative risk of recurrence. As expected, higher values for this relative risk lead to higher point prevalence values. As with the other Figures, the data points in the Figure are based on n=25 replications.

Figure 11. Simulated Point Prevalence, by Values for Relative Risk of Recurrence.

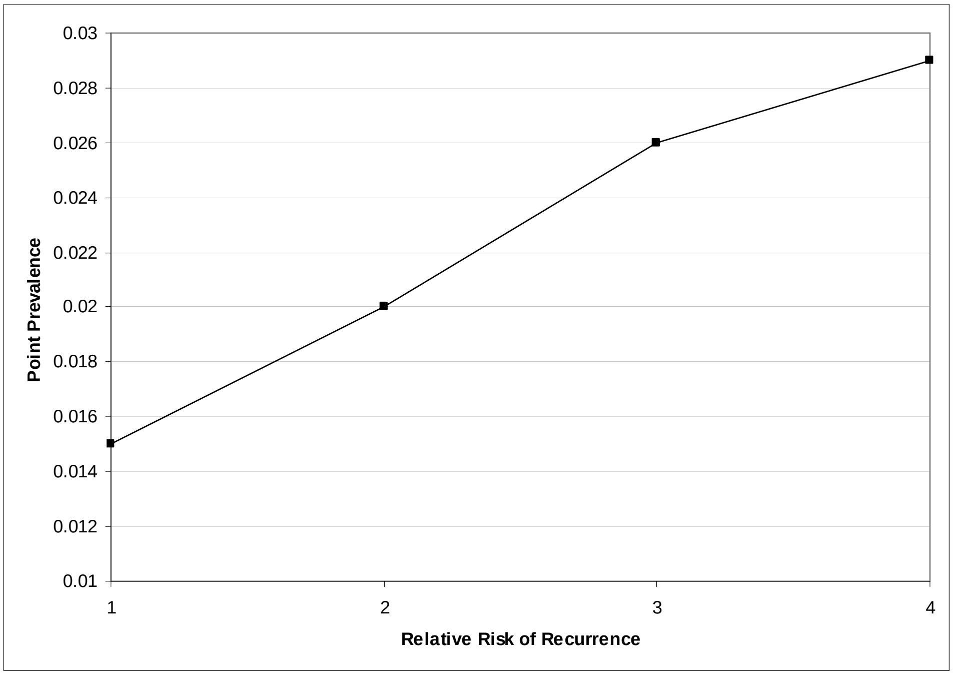

Figure 11 presages one of the important implications of the simulation studies presented in this paper. As point prevalence is strongly linked to the relative risk of recurrence in a group with highly recurrent major depression, health care strategies that target this variable are promising options for reducing disease burden in the population. This subset of people with major depression represents the group who are candidates for long-term treatment.

Mortality

In the main simulation study, a value of 1.1 was selected for the relative risk of mortality for people with major depressive disorders (one or more lifetime episodes of major depression). Mortality is a determinant of prevalence, and expectation holds that if

this relative risk value were to increase, the prevalence of major depression will decrease. Figure 12 presents simulated point prevalence of major depression across a range of values for the relative risk of mortality, confirming this expectation.

Figure 12. Simulated Point Prevalence of Major Depression, by Relative Risk for Mortality.

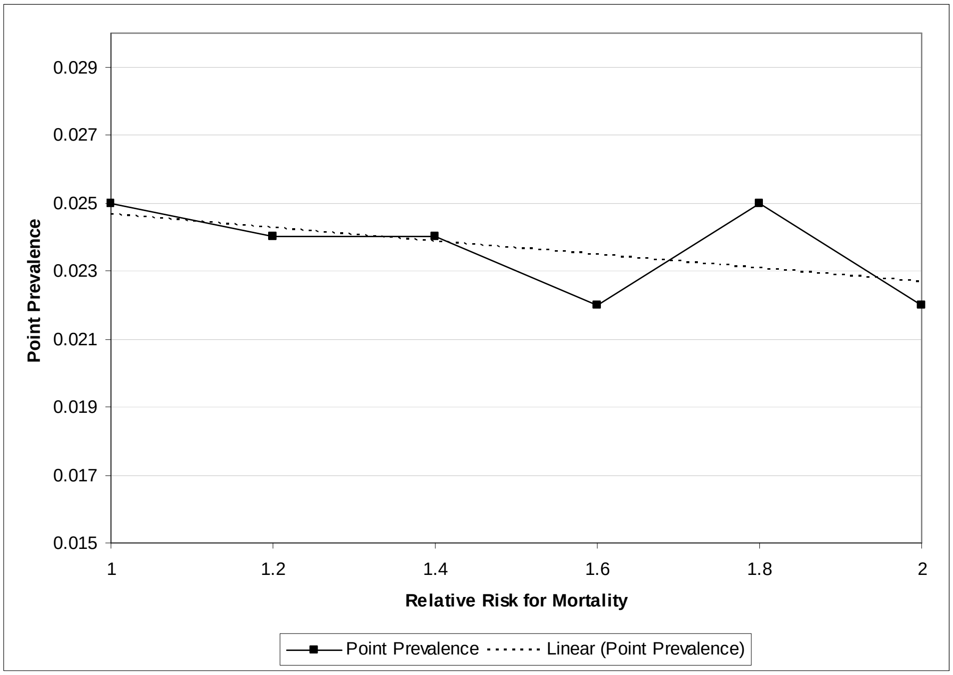

Episode Duration

This variable was also represented using a Weibull distribution. Here, the distribution represents time to recovery. A larger scale parameter indicates a longer time to recovery, so increasing the size of this parameter is expected to result in higher

simulated period prevalence. This expectation was confirmed in a series of replicated (n=25) simulations, see Figure 13.

Figure 13. Simulated Point Prevalence, by Scale Parameter for Episode Duration (Shape Parameter = 0.521).

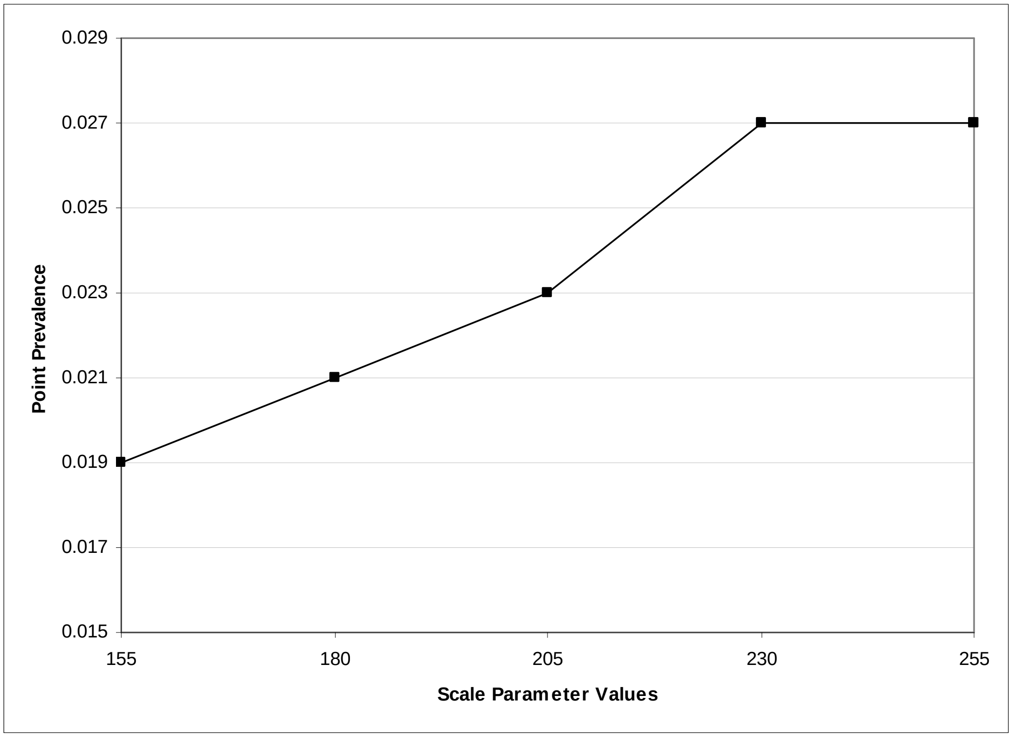

Increasing values of the shape parameter for episode duration mean that the recovery rate will not decline as rapidly over time, leading to episodes that are, on average, more brief. In turn, this should lead to lower prevalence, as depicted in the series of simulations presented in Figure 14.

Figure 14. Simulated Point Prevalence, by Shape Parameter for Episode Duration (Scale Parameter = 205).

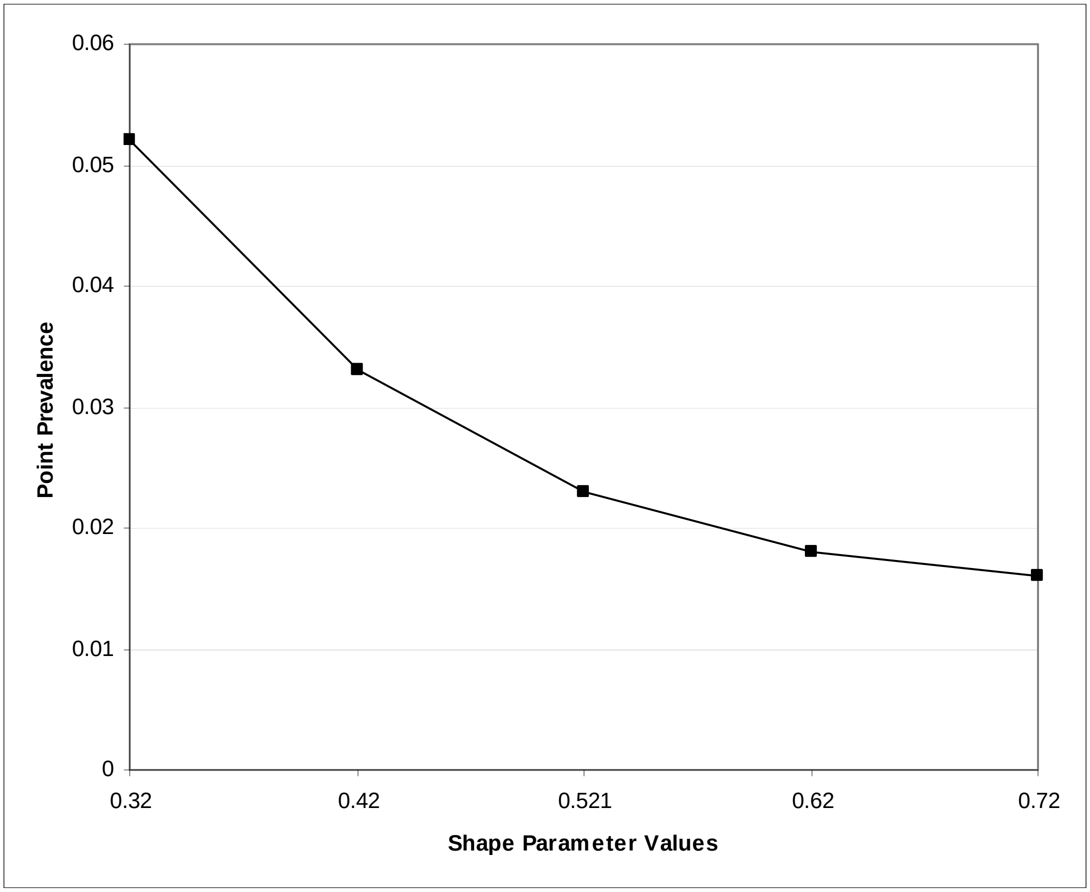

Supplement: Additional file 2 [file 1471-244X-7-23-S2.pdf]
